# Multiobjective Fusion Decision Model for Key Technologies of Aircraft Intelligent Lightweight

**DOI:** 10.1155/2022/1447718

**Published:** 2022-02-15

**Authors:** Anliang Zhou, Lei Dong, Yuedong Lang, Jiangping Cui, Wei Ren, Jiankang Liu, Zhiqiang Wang

**Affiliations:** ^1^School of Mechanical and Electrical Engineering, Beijing Institute of Graphic Communication, Beijing 102600, China; ^2^School of Mechanical Engineering, Tianjin University of Technology and Education, Tianjin 300222, China; ^3^Beijing Aerospace Control Instrument Research Institute, Beijing 100854, China; ^4^China Mobile Procurement Shared Service Center, Beijing 102200, China; ^5^Beijing Readir Technology Co., Ltd, Beijing 100031, China

## Abstract

As a tool for aviation exploration, aircraft plays a pivotal role in the aerospace field. The aircraft's lightweight can ensure that the aircraft can carry more loads, so it is of great significance to the research on the key technologies of aircraft lightweight. This paper organizes the intelligent technology system from the multiobjective fusion technology relationships and considers the complex changes between technology units. A technology group model for the intelligent technology system of spacecraft welding is formed to provide a decision-making basis for the realization of batch, stable, and efficient intelligent and lightweight manufacturing of spacecraft. We expand the technology development research from single technology research to technology system research. We research and build a three-dimensional perspective intelligent technology system based on technology unit, technology system, and technology group. It reveals the internal relationship description of technological development and expands and perfects the current research theory of technological development.

## 1. Introduction

Aircraft has been widely used and plays a pivotal role in the aerospace field [1–3]. As one of the key technologies in aircraft research and development, lightweight design has received extensive attention [4, 5]. Currently, intelligent technologies related to lightweight are being deeply integrated with traditional manufacturing processes and accelerate the conventional manufacturing process and development mode [6, 7]. It is triggering a new round in lightweight technology and industrial development models [8]. This change is reflected in increasingly lightweight designs that entirely apply intelligent technology to achieve an all-round digital and intelligent transformation of the manufacturing process, research and development, production, management, and service [9]. The quality of aircraft products and the production efficiency have been continuously improved. It has driven the rapid development of many related intelligent technologies, intelligent equipment, and intelligent products, providing necessary manufacturing process support for the manufacturing industry [10, 11]. Therefore, the integration of lightweight and intelligent technology is of great significance for the development of aircraft intelligent manufacturing.

In recent years, many scholars have done a lot of research on lightweight technology. To sum up, it mainly includes the following three aspects: lightweight design technology, lightweight material technology, and lightweight manufacturing technology. Wu et al. [12] and Fiedler et al. [6] achieved the effect of lightweight design through topology optimization design under the premise of satisfying safety. Zuo [7] carried out structural optimization under the constraints of static stiffness and dynamic frequency stiffness and achieved good lightweight effect. Cho et al. [13] used the size optimization method to optimize the design of the rail transit car body and achieved a lightweight effect by reducing the body weight by 29%. Hou et al. [14] optimized the multiobjective design of structural performance, cost, and mass and reduced the mass of the components while increasing the structural stiffness. Emirci and Yildiz [15] compared and analyzed the anticollision and energy-absorbing characteristics of the body energy-absorbing box using aluminium alloy and high-strength steel. Through lightweight material technology, the aluminium-made energy-absorbing box is better than the energy-absorbing effect per unit mass and the target of the steel energy-absorbing pack. Parsa and Darbandi [16] analyzed the application of hydroforming in car body forming and achieved lightening from the manufacturing technology.

Although the above lightweight methods have achieved good results, the applied methods are relatively single, and the influence of the mutual coupling of multiple factors has not been considered. For aircraft, various factors such as product quality, performance, and manufacturing cycle require higher lightweight technology [17]. Especially in the welding process of lightweight materials, it is urgent to change the current situation of unstable quality of welding products and dependence on operator skills [18–20]. The application of intelligent digital technology and lightweight knowledge base can realize the optimization of welding process parameters and the identification of welding defects [21, 22]. At the same time, uncontrollable factors such as welding cracks and deformation are eliminated, welding accuracy is improved, the welding production mode is changed, and high-quality and high-efficiency manufacturing of lightweight material welded products is supported [23]. To better combine intelligent technology with lightweight goal, it is necessary to clarify the correlation factors between lightweight technology and system analysis method. At the same time, a set of technical systems is formed according to its technical units, and a multiobjective fusion decision-making model is established. It will be of great significance to describe the development of technology units in the way of technology group [24–26].

This paper presents a multiobjective fusion decision model applied to the key technology of spacecraft. The model fully considers the complex changes between technical units, organizes the intelligent technology system, and forms a technology group for spacecraft intelligent lightweight technology system. The research on the development of lightweight technology is expanded from single technology research to the technical system of the connection between technical units. The three-dimensional perspective intelligent technology system of technology unit, technology system, and technology group is constructed. It provides a decision-making basis for spacecraft batch, stable, efficient, and lightweight manufacturing.

## 2. Concept and Method

### 2.1. Mathematical Representation of the Technical System

The technology system construction method aims to reveal the specific relationship research object of the intelligent technology unit set to the technology system with specific relationships (defined as). The technology group (defined as) can be described as a set of specific relationships. The corresponding representations of the three are as follows:

  Technical unit set: Y={1,2,3, ⋯y, }
  Technical system set: P={Y_1_, Y_m_, Y_n_, ⋯} 
  Technology cluster: T={T_1_, T_m_, T_n_, ⋯} , T for a specific connection

The system construction of technology development can be described as defining a specific relationship *T* on the set *Y*, which describes the specific relationship from the technology unit set *Y* to the case set *S*, which is described as *T* ⊆ *Y* × *S*. That is, the relationship *T* = {*Y*, *T*_c_} is defined on the technical unit set and the relationship *T* = {*S*, *T*_*Q*_} is defined on the case set, so *T* describes *T*, respectively, *T*_C_ and *T*_Q_ define two specific relation operations on sets Y and S, so that a group of things with regular interaction or interdependence form a whole, that is, the technical system P, P is described as the abstract system subset *P* : *T* ⊆ *Y* × *S* ⟶ *Y* formed by the relation operation from the technical unit set Y to the specific relation *t* of the case set *S* × *S* ⟶ Y. The specific relationship of S is the mapping from *T* to *Y*. Furthermore, the technical system *P* is described as *Y*, × , *S*. That is, the technical system P is a subset of the specific relationship *T* from the technical unit set *Y* to the case set *S*.

### 2.2. Unit Analysis of the Technical System

**Figure 1:**
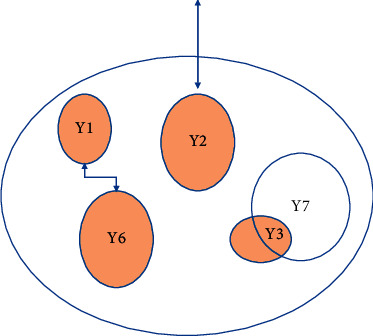
Evolution of technology unit relationship.

Make a good selection of technical units with specific internal relations to form a technical system around internal ties. Several technical systems form a technical group with specific internal ties. This method includes a dynamically related technical unit technical system-technical group to describe the relationship between the development of intelligent technology. Based on technical units, the technology group and technology system for decision-makers can be optimized, and the technology system can be adjusted and optimized through the technology group, or the new technical units can be expanded and supplemented to form a set of dynamic evolution correspondence. The technical system composes technical units, and the technical units are continuously developed and evolved. For a specific technical unit of a system, it will not only be affected by other technical units in the system but also by changes in the external environment and be reorganized with changes in the objectives of the technical system, as shown in Figure 1.

Y1 The technical complexity of Y2 and Y3 is different. For example, the technical system composed of special alloy precision casting technology, comprehensive design optimization technology, and virtual manufacturing technology based on product life cycle management describes the technical system of special alloy precision casting facing the full life cycle of products with different dimensions of process, process design and process production management, and the comprehensive design optimization technology refers to special alloy. In the design stage of gold casting products, the technology or technical complexity required is different from the whole life cycle management of precision casting equipment and the manufacturing process. Similarly, the coverage and complexity of special alloy precision manufacturing equipment technology, comprehensive design optimization technology, and life cycle management technology of manufacturing process are also different. At the same time, the comprehensive design optimization technology has many common applications, which can be used in other technical systems outside the technical system and can also improve its design optimization ability and methods due to the changes of the external development environment, the comprehensive optimization design technology itself also needs to consider the ability of special alloy precision casting technology. The comprehensive optimization design beyond the capability is not supported by special process equipment for equal material manufacturing. The two are combined, as shown in Figure units *Y*3 and *Y*7. Product life cycle management technology can be used both inside and outside the technical system. As shown in Figure 1, *Y*2 technical units will also be greatly affected by changes in technical means or environment outside the technical system.

**Figure 2:**
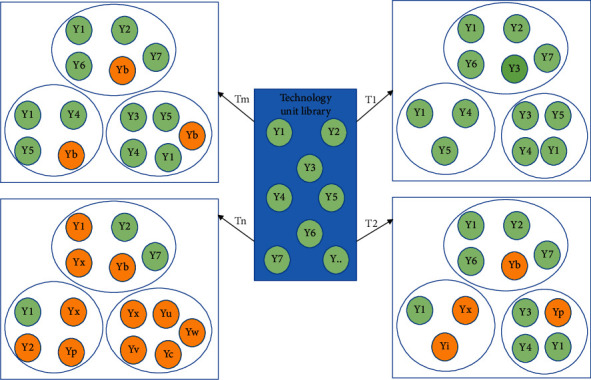
Technical system construction diagram of multitarget fusion.

The specific relationship between *Y*1 and *y*6 and *Y*3 and *Y*7 described in the technical unit also has the difference of sequential relationship and cross-relationship. For example, the technical system is composed of welding process real-time monitoring and detection technology, welding defect automatic identification technology, high-precision welding robot, and welding power supply. The welding process real-time monitoring and detection technology are the premise of welding defect automatic identification technology. With the welding process real-time monitoring and detection technology, the welding defect automatic identification technology can be carried out. The two restrict each other, appear in a sequence, and work together, as shown in Y1 and y6 in Figure 2. The high-precision welding robot and welding power supply are closely connected, overlapped, and crossed to form an association relationship between connecting electric energy and forming a stable execution action, as shown in Y3 and Y7 in Figure 1. The diagram only shows the order and cross between technical units, and there may be more connections.

*Y*2 described in the technical unit interacts with the outside of the technical system, which may be affected by the external technical system or environment or may affect the external technical system or environment. For example, the welding expert system *Y*2 will not only affect the welding production system but also affect the welding production process layout. In addition, the technical system is also affected by process objectives or value objectives, and even the changes of objectives and systems caused by changes in the external environment should be considered. For example, there are differences in the correlation model of technical units of a welding system aiming at high precision or high efficiency; for another example, for a certain product, whether the market is easy to accept personalized customization or more inclined to mass manufacturing also has a great impact on the technical system.

### 2.3. Correlation Analysis of the Technical System

The technology system is composed of technology units with specific association relations. Different association relations have a great impact on the technology system. There are many kinds of association relationships. For example, there is a relationship between process and function between product process and intelligent function. There is a relationship between product quality improvement and production line production efficiency, stability, and consistency, as well as process optimization and automation improvement. As shown in Table 1, List *T*1 (production line production efficiency), *T*2 (intelligent smelting system), and T3 related to its technical unit (equipment fault prediction and diagnosis) is a specific correlation. Based on the listed correlations, the technical unit forms a specific target-oriented technical system or technology group. It describes the technical systems of the four types of correlation on the right side of the table. Due to the correlation, the orderly combination of multiple technical units is realized to form the technical system described in the table. The correlation of the technical system is the decisive factor of organizing the technology units and is also the goal of the formation of the technology system.

### 2.4. Technical System Construction of Multiobjective Fusion

In the construction of the technical systems, we often consider the fusion scheme aiming at a variety of correlation relations and comprehensively consider the complex changes between technical units to form a multiobjective fusion technical system [27–29]. It is precise because of the complexity of the integration of multiple relationships and the complexity of the relationship between technical units that constitute the complexity and uncertainty of the technical system. The technical systems constructed are different due to different technical associations *T*1 and *T*2; due to other fusion targets TM and TN, other technical systems are built, as shown in Figure 2.

Figure 2 reveals the complexity and diversity of intelligent technology systems. It can be seen from the diagram that the cluster T={T_1_, T_m_, T_n_, ⋯}. The research of technology connection based on the technology unit library has become an eternal theme. It can also be considered that the research on the development of intelligent technology focuses on the change of technology units and technology connections. The change of technology units, technology connection, and the evolution of external environment of technology system constitute a complete system of technology development.

## 3. Multiobjective Fusion Decision-Making Model

**Figure 3:**
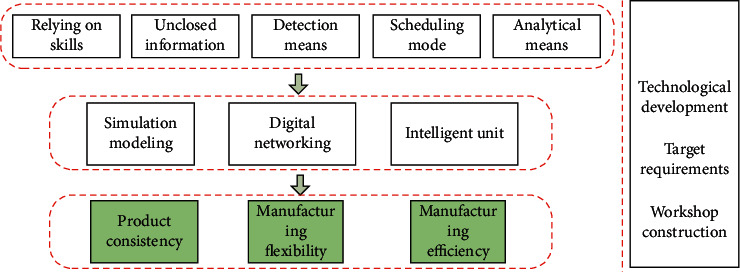
Element Atlas of lightweight material welding workshop.

Because of the above specific problems of the aircraft welding process, the key to the development of intelligent technology lies in the intelligent improvement of the quality, performance, and manufacturing cycle of relevant products, that is, the target relationship of aircraft lightweight material welding technology system. The traditional lightweight material welding workshop has some prominent problems. Product quality control does not form a closed loop, process design depends on human experience, low degree of equipment automation, backward detection means, and so on. There is an urgent need to transform and upgrade to the intelligent direction to achieve the synchronous improvement of product consistency, manufacturing flexibility, and efficiency. A multiobjective fusion relationship has been formed for such problems, then selecting relevant technology units from the Technology Library and external technology environment, as shown in Figure 3.

Based on the final goal and demand of lightweight material welding, combined with the technical unit library, seven intelligent technology systems P={Y_1_, Y_2_,…, Y_7_} for the intelligentization of lightweight material welding workshops are analyzed and the following seven major process problems are solved, respectively:

 
*P* = {
  Question 1: describe the welding method, process parameters, and scope of the configuration of the welding equipment, drive, and control system.
  Question 2: establish a database to store and compile the data of various existing welding production processes for sharing by all welding workstations.
  Question 3: according to the welding method, welding material types, and specifications and other data, formulate a program for optimizing welding process parameters.
  Question 4: according to the workpiece shape, size, and the preset deviation limit of the seam, the automatic correction and compensation program are compiled.
  Question 5: according to the image of the welding area, according to the shape of the welding arc and the welding bead, the image recognition, and remote monitoring program are compiled.
  Question 6: prepare the automatic fault diagnosis, alarm, and repair program for the drive system and control system of welding equipment, wire feeder, and welding power source.
  Question 7: manage, distribute, and collect the data between the welding equipment control system and the DCS control system operating station and realize the monitoring and remote diagnosis functions of the processing process.
  }

The corresponding eight technical systems are as follows:

Y1 refers to rapid interchange clamping (flexibility). Y2 refers to the instrumentalization of weld quality inspection. Y3 refers to the digitization of welding production management. Y4 refers to the real-time management of welding control. Y5 refers to the paperless basic welding database. Y6 refers to the computerization of weldability analysis. Y7 refers to the intellectualization of welding process analysis.

From the dimensions of different fusion associations, the decision model for the association between technical units-technical systems-technical groups is described as follows.

From the shop floor construction and target demand dimension. DCS unified the control and deployment of the intelligent welding workshop, the production tasks issued by the management, the manufacturing unit prefabricated action program, and test data according to the instructions to determine the entire manufacturing process. Everything is ready, the work program starts the welding command, and the main control unit collects the processing parameters in real time. At the same time, online inspection, quality control, welding process monitoring, analysis, and process optimization and adjustment are carried out from the dimension of welding technology development. Intelligent welding technology effectively improves the accuracy and reliability of product quality. Focusing on the continuous improvement of welding control technology such as welding quality, welding efficiency, and welding spatter, foreign manufacturers have taken the lead in realizing the application of full digitalization in welding equipment, including the precise operation control and working process control of the microprocessor to achieve various welding performances. These digital and intelligent applications make welding integrated and simplified, no more reliance on welders and traditional welding processes. At the same time, in a concise time, the change of arc length can be controlled accurately and reliably. The software upgrade of the welding equipment can also be applied to the needs of different occasions to ensure the beauty of the welding seam. Due to the precise computing capability of the microprocessor, various welding specifications and precise arc starting and ending can be achieved.

Therefore, the aircraft welding workshop is applied to seven technical systems through the decision model with the dimensions of different correlation and technology development, integration, quick interchange clamping (flexibility), totalization of weld quality inspection, vitalization of welding production management, real-time welding control management, closed-loop welding process quality control, paperless welding basic database, datalization of weldability analysis, and intellectualization of welding process analysis. It is precisely because of the application of the technology group formed by the construction of these eight technical systems that the batch, stable, and efficient manufacturing of aircraft is realized manufacturing capacity.

## 4. Results and Discussion

**Figure 4:**
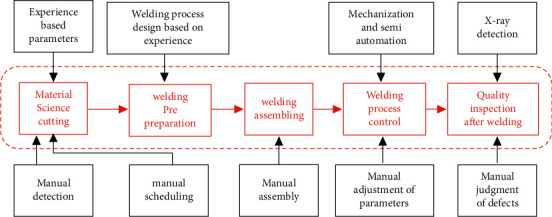
Manufacturing technology means of the current welding workshop.

To further improve the production capacity and technical level of the welding process of aircraft lightweight materials, China has carried out a series of intelligent technology innovations for domestic aircraft manufacturing enterprises with the support of 04 unique project. The technical means adopted for the welding process of lightweight aircraft materials are shown in Figure 4.

The main problems existing in the welding of lightweight aircraft materials can be summarized into the trial set to be solved by the technology group, that is, the correlation set:

 
*T* = {
 
*T*1: traditional methods based on manual and experience are primarily used in auxiliary links such as clamping and measurement, automatic welding, parameter selection, and process control. There are still difficulties of insufficient product quality stability and low efficiency.
 
*T*2: product quality depends on the skills of skilled workers, and there is no closed loop of product quality control, and the stability of product quality is difficult to control.
 
*T*3: restricted by the relatively backward parameter, optimization level and detection methods, defect determination, scheduling method, clamping methods, and other factors, the demand for fast and efficient manufacturing is difficult to achieve.
 
*T*4: product manufacturability analysis still relies on personal knowledge and experience. The flexibility of product welding and manufacturing and the ability to quickly replicate the production line cannot meet the actual needs.
  }

First, the leading technical units in lightweight material welding are analyzed. Manufacturing enterprises in automobiles and airplanes in industrialized countries have widely applied automation technology, sensor technology, intelligent technology, information technology, robot technology, etc., and gradually realized the digitization and intelligence of manufacturing workshops, representing a higher level of development of manufacturing workshops. Among them, the more typical technical groups influencing factors for the development of technical units are the following: (1) The rapid development of products or equipment is realized by digital modelling and simulation of products and workshops, and some products or components have been successfully developed, shortening the product development cycle to achieve rapid response manufacturing. (2) Through the upgrade of the digital network of the manufacturing site, to realize the monitoring of equipment operating parameters in the production process of the enterprise and the process monitoring of production system failures. (3) Realize workshop intelligence with the precision and intelligence of manufacturing equipment units.

Second, combined with the relationship of multiobjective fusion of aircraft, the improvement of welding manufacturing efficiency, manufacturing flexibility, and product consistency of lightweight materials can be achieved. The intelligent welding workshop adopts advanced technical means such as intelligent manufacturing unit, simulation modelling, and digital networking. To solve various existing problems. Through the intelligent upgrade, the elements of technology development dimension, target demand dimension, and workshop construction dimension are satisfied. It is also the influencing factor of the technology group in the development of the technology unit.

Finally, the influence of technology group development is analyzed from the perspective of welding technology and process function. The technical units of the unit library of multiple welding workshops are selected for analysis. Taking a typical welding workshop as an example, its process and corresponding technical units are described as follows: (1) Define the welding method, process parameters, and scope. (2) A database is established to store the data of various welding production processes and shared by all welding workstations. (3) According to the data of welding method, welding material type and specifications, etc., formulate a program for optimizing welding process parameters. (4) According to the workpiece shape, size, and the preset deviation limit of the seam, the automatic correction and compensation program are compiled. (5) According to the real-time recording of the welding area image during the welding process, the remote monitoring program is compiled according to the welding arc and welding beam shape parameters. (6) Set up automatic fault diagnosis alarm and repair procedures, which are applied to the driving system and control system of welding equipment, as well as wire feeder and welding power supply. (7) Manage, distribute, and collect the data between the welding equipment control system and the DCS control system operating station and realize the monitoring and remote diagnosis functions of the processing process.

## 5. Conclusions

This article proposes a multiobjective fusion decision-making model for aircraft intelligent lightweight key technologies. The model fully considers the complex changes between technical units and describes the development of technical units in a technical system in the form of technical groups. Extend the lightweight technology from single technology research to a technology system that studies the connection between technology units. In this paper, a technical unit technical system-technical group three-dimensional perspective intelligent technology system is constructed. It reveals the description of the internal connection of technology development and expands and perfects the current research theory of technology development. And it provides a decision-making basis for the batch, stable, efficient, and lightweight manufacturing of spacecraft. At the same time, the model can further expand the scope of industrial applications, which is very beneficial for improving the decision-making method of various equipment and systems' lightweight and provides a powerful tool for the lightweight design of various equipment.

To sum up, this paper studied the multiobjective of the key technology of aircraft intelligent lightweight and proposed a multiobjective fusion decision-making model. Due to the limitations of experimental conditions, time, and energy, the model needs to be further improved: first, the key technologies of intelligent lightweight cover a wide range, and the factors are influenced and coupled with each other, and it is difficult to cover the expert knowledge fully. The selected experimental cases and technical analysis are inevitably one-sided and limited, so it is necessary to improve the model further. Second, due to the small number of aircraft samples, the selected technical units, technical systems, and technical groups have limitations in the stereoscopic perspective. Further analysis, mining, and improvement are needed to increase the scope of application.

## Figures and Tables

**Table 1:** Example of system association relationship construction.

Number	Association relationship	Technical system example
*T*1	Production efficiency of the welding production line	Intelligent welding process control: welding seam forming control technology based on visual sensing, constant pressure adaptive control technology, welding path adaptive control technology, and arc length adaptive control technology based on laser sensor tracking. These four central technical units constitute a technical system for realizing the automatic control of welding path and welding process parameters and improving the automatic control level of the welding process.
*T*2	Casting melting intelligent system	Casting intelligent melting system: it is composed of intelligent feeding and unloading technology, online detection technology of molten iron parameters, LIMS (Laboratory) information management technology, and intelligent 3D printing technology of casting mould. These four main technical units constitute the technical system to cast the intelligent melting.
*T*3	Fault prediction and diagnosis of forging equipment	Equipment fault prediction and diagnosis: it comprises equipment fault diagnosis system technology, product data acquisition technology using RFID, QR code/bar code, intelligent control, intelligent decision support, and visualization tool technology. These three main technical units aim at equipment fault prediction and diagnosis.

## Data Availability

The data used to support the findings of this study are included within the article.
